# Quantum simulation of particle pair creation near the event horizon

**DOI:** 10.1093/nsr/nwaa111

**Published:** 2020-05-30

**Authors:** Yao Wang, Chong Sheng, Yong-Heng Lu, Jun Gao, Yi-Jun Chang, Xiao-Ling Pang, Tian-Huai Yang, Shi-Ning Zhu, Hui Liu, Xian-Min Jin

**Affiliations:** Center for Integrated Quantum Information Technologies (IQIT), School of Physics and Astronomy and State Key Laboratory of Advanced Optical Communication Systems and Networks, Shanghai Jiao Tong University, Shanghai 200240, China; National Laboratory of Solid State Microstructures and School of Physics, Collaborative Innovation Center of Advanced Microstructures, Nanjing University, Nanjing 210093, China; Center for Integrated Quantum Information Technologies (IQIT), School of Physics and Astronomy and State Key Laboratory of Advanced Optical Communication Systems and Networks, Shanghai Jiao Tong University, Shanghai 200240, China; Center for Integrated Quantum Information Technologies (IQIT), School of Physics and Astronomy and State Key Laboratory of Advanced Optical Communication Systems and Networks, Shanghai Jiao Tong University, Shanghai 200240, China; Center for Integrated Quantum Information Technologies (IQIT), School of Physics and Astronomy and State Key Laboratory of Advanced Optical Communication Systems and Networks, Shanghai Jiao Tong University, Shanghai 200240, China; Center for Integrated Quantum Information Technologies (IQIT), School of Physics and Astronomy and State Key Laboratory of Advanced Optical Communication Systems and Networks, Shanghai Jiao Tong University, Shanghai 200240, China; Center for Integrated Quantum Information Technologies (IQIT), School of Physics and Astronomy and State Key Laboratory of Advanced Optical Communication Systems and Networks, Shanghai Jiao Tong University, Shanghai 200240, China; National Laboratory of Solid State Microstructures and School of Physics, Collaborative Innovation Center of Advanced Microstructures, Nanjing University, Nanjing 210093, China; National Laboratory of Solid State Microstructures and School of Physics, Collaborative Innovation Center of Advanced Microstructures, Nanjing University, Nanjing 210093, China; Center for Integrated Quantum Information Technologies (IQIT), School of Physics and Astronomy and State Key Laboratory of Advanced Optical Communication Systems and Networks, Shanghai Jiao Tong University, Shanghai 200240, China; CAS Center for Excellence and Synergetic Innovation Center in Quantum Information and Quantum Physics, University of Science and Technology of China, Hefei 230026, China

**Keywords:** quantum simulation, general relativity, transform optics, particle pair creation, black hole

## Abstract

Though it is still a big challenge to unify general relativity and quantum mechanics in modern physics, the theory of quantum field related with the gravitational effect has been well developed and some striking phenomena are predicted, such as Hawking radiation. However, the direct measurement of these quantum effects under general relativity is far beyond present experiment techniques. Fortunately, the emulation of general relativity phenomena in the laboratory has become accessible in recent years. However, up to now, these simulations are limited either in classical regime or in flat space whereas quantum simulation related with general relativity is rarely involved. Here we propose and experimentally demonstrate a quantum evolution of fermions in close proximity to an artificial black hole on a photonic chip. We successfully observe the acceleration behavior, quantum creation, and evolution of a fermion pair near the event horizon: a single-photon wave packet with positive energy escapes from the black hole while negative energy is captured. Our extensible platform not only provides a route to access quantum effects related with general relativity, but also has the potentiality to investigate quantum gravity in future.

## Teaser

We demonstrate a quantum evolution of fermions in close proximity to an artificial black hole on a photonic chip, providing a route to access quantum effects related with general relativity and the potentiality to investigate quantum gravity in future.

## INTRODUCTION

Quantum gravity is a great challenge field in modern physics which seeks to describe gravity based on the principles of quantum physics and finally needs unification of quantum mechanics and general relativity. A number of quantum gravity theories, such as string theory and loop quantum gravity, have been proposed to fill the long-outstanding gap. However, there is still no complete and consistent quantum theory of gravity, and these candidate models still need to overcome major formal and conceptual problems. Nevertheless, the flourishment of quantum field theory in curved spacetime can describe gravity where quantum effects cannot be ignored, such as Hawking radiation [1] which happens near compact astrophysical objects possessing strong effects of gravity. Owing to the limitation of cosmological observation, however, the possibility to directly measure these effects is far beyond present experiment technique. Fortunately, analog models from various systems in the laboratory environment have been motivated by the ability of investigating phenomena not readily accessible in the cosmological counterparts. Examples are Hawking–Unruh radiation emulated from a Fermi-degenerate liquid [2], a superconductor [3], optical fiber [4,5], nonlinear crystal [6], ion ring [7], and Bose–Einstein condensates [8].

On the other hand, transformation optics [9–12] that manipulate permittivity and permeability profiles of materials can be extensively investigated to design many artificial systems with novel optical applications [13–21]. One fascinating example is invisibility cloak [13–19], in which light is regarded as linear parallel geodesic rays in deformed spaces. Recently, through mapping the metric of spacetime to electromagnetic-medium constitutive parameters with local curvature, it has become possible to mimic general relativity phenomena. Examples from general relativity are black holes [22–27], Einstein ring [28], cosmic string [29–31], Minkowski spacetimes [32], wormholes [33–35], De-Sitter Space [35], cosmological inflation, and redshift [36]. The underlying principle is the form invariance of Maxwell’s equations between the complex inhomogeneous media and the background of an arbitrary spacetime metric. Furthermore, some other optical structures, such as curved waveguides [37,38], nonlocal media [39,40], meta-chains [41], and metasurfaces [42], have been harnessed to mimic the gravitational effects. In addition, coupled waveguides arrays [43,44] also allow for the optical simulation of relativistic phenomena, such as *Zitterbewegung* [45], Klein tunneling [46], and neutrino oscillations [47] as well as fermion pair production [48,49]. However, all these works are limited either in classical regime or in the flat space, leaving the quantum evolution related with general relativity uninvestigated.

In this work, we propose and realize the quantum evolution of single-photon wave packet close to the event horizon of an artificial black hole on a photonic chip, in which the coupled three-dimensional waveguide arrays are fabricated by femtosecond laser direct writing technique [50,51]. Due to the high controllability over the coupling and the on-site energy among waveguides in a physically scalable three-dimensional structure [52,53], one-dimensional artificial black holes can be constructed using single-layer photonic waveguide lattice with inhomogeneous hopping coefficients inspired by the concept of transformation optics. Comparing to linear time evolution in the flat space, the dynamic behavior of single-photon wave packet near the event horizon of the black hole has an exponential form as time, whose exponential index depends on the curvature of the black hole. We also experimentally observe the acceleration behavior, creation and evolution of fermion pair close to the event horizon of an artificial black hole by using a designed bi-layer photonic waveguide lattice: a single-photon wave packet with positive energy escapes while negative energy is captured. This phenomenon deviates from the intuition that photon is always trapped by the black hole, and is found well analogue to Hawking Radiation, which completely origins from quantum effects related with gravitational effect. Owing to vacuum fluctuations, particle–antiparticle pair is generated close to the event horizon of a black hole. One of the particles with negative energy falls into the black hole while the other escapes. This causes the black hole to lose mass, and it appears that the black hole has just emitted a particle. Besides studying quantum field theory related with gravitational effect, our platform also has the potentiality to investigate quantum gravity.

## RESULTS AND DISCUSSION

### Constructing artificial black holes on a photonic chip

To study the quantum evolution of boson in close proximity to a black hole, we start with a spacetime metric for a two-dimensional Schwarzschild black hole near the horizon:

(1) \begin{equation*} d s^{2}=\alpha ^{2} r^{2} d t^{2}-d r^{2}, \end{equation*}

where α = 1/2*r*_*s*_ is the space curvature, *r*_*s*_ = 2*M* is the radius of the black hole, *M* is the mass of the black hole, and nature units have been adopted (*G* = *c* = 1). When considering boson motion, it satisfies null geodesic *ds* = 0, then we obtain the evolution function as *r* = *r*_0_ exp (α · *t*), where *r*_0_ is initial position. The result clarifies that the quantum evolution near the event horizon of a black hole is faster than that in flat space, and the exponential evolution index depends on the curvature of the black hole α.

**Figure 1.**
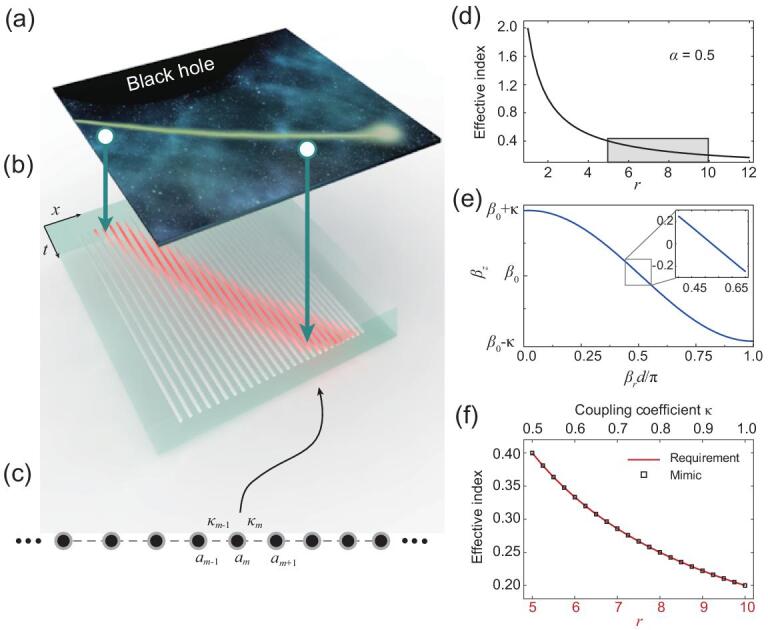
Constructing an artificial black hole on a photonic chip. (a, b) The schematic of mapping the event horizon of a black hole to the photonic waveguide lattice. The motion of particle near the black hole (a) can be mapped to the transfer of a single-photon wave packet on a photonic lattice (b), where the longitudinal direction of waveguide maps the time while the transverse maps the space. (c) The schematic of the photonic lattice. The evanescently coupled photonic waveguide lattice owns inhomogeneous hopping coefficients as κ_*m*_ = α*m*/2 and the corresponding black hole lies in the left side. (d) The required effective index for constructing the event horizon of the black hole in transformation optics. (e) The relationship between transverse and longitudinal wavevector of the photonic waveguide lattices. The inset shows that the linear relationship between β_$z$_ and β_*r*_ around β_*r*_ = π/2*d*. (f) The comparison between the required effective index of the event horizon of the black hole and the mimic by controlling coupling coefficients of photonic lattice.

To mimic the event horizon of an artificial black hole on a photonic chip as shown in Fig. 1a–c, we borrow the concept of transformation optics, that the propagation of electromagnetic waves in static gravitational field is analogue to the propagation in inhomogeneous medium. As is shown in Fig. 1d, the evolution of photon in a gravitational field behaves similarly as in an optical media with an effective refraction index [22]:

(2) \begin{equation*} n=\sqrt{-g_{11} / g_{00}}=1 / \alpha r, \end{equation*}

where *g*_00_ = α^2^*r*^2^, *g*_11_ = −1. To experimentally obtain the inhomogeneous effective refractive index that corresponds to Schwarzschild black hole, we exploit evanescently coupled waveguides with inhomogeneous hopping coefficients, which are fabricated in three dimensions by using femtosecond laser direct writing technique. The dynamics of photon in the photonic lattice can be described by a set of coupled discrete Schrodinger equations, which are derived from Schrodinger-type paraxial wave equation by employing the tight-binding approximation: *i*∂ϕ_*m*_/∂$z$ = β_0_ϕ_*m*_ − κ_*m*−1_ϕ_*m*−1_ − κ_*m*_ϕ_*m*+1_, where ϕ_*m*_ is the complex field amplitude of site *m*, $z$ is the propagation distance along the waveguides mapping the time variable, β_0_ is on site energy of each waveguide, parameter κ_*m*_ represents the coupling strength between the adjacent sites.

Taking coupling coefficient as κ_*m*−1_ = κ_*m*_ = κ and substituting the plane wave solution ϕ_*m*_ ∝ exp (*i*β_*r*_*md* + *i*β_$z$_$z$), we can obtain the dispersion relation that connects transverse and longitudinal dynamics: β_$z$_ = β_0_ − 2κcos (β_*r*_*d*), where β_*r*_ and β_$z$_ are transverse and longitudinal wavevector, respectively, *d* is waveguide spacing (see Fig. 1e). After photon evolves over distance Δ$z$, each transverse component gains a phase Φ = β_$z$_(β_*r*_)Δ$z$, and then the corresponding transverse shift centered around β_*r*_ is Δ*r* = ∂Φ/∂β_*r*_ = (∂β_$z$_/∂β_*r*_)Δ$z$. Since the propagation distance $z$ in the coupled waveguide equation plays the role as the time *t* in Schrodinger equation, we can define the velocity of wave packet in such system as $v$ = Δ*r*/Δ$z$ = ∂β_$z$_/∂β_*r*_ = 2κ*d*, when exciting the coupled waveguide with the transverse wavevector as around β_*r*_ = π/2*d*. The effective refractive is then defined as *n* = 1/$v$ = 1/(2κ*d*) (see Supporting Information I for details). Comparing with Eq. (2), the effective refractive index required by one-dimensional black hole will be identical when we control the coupling coefficient as κ = α*r*/(2*d*) = α*m*/2, where we take discrete the continuous function with lattice *r* = *md*. Therefore, by constructing the coupling coefficient κ increasing linearly with waveguide site *m* as shown in Fig. 1f, we can achieve the required effective refractive index and the single-photon wave packet can simulate the quantum evolution of boson near the event horizon of the black hole.

### Experimental quantum evolution of boson in close proximity to a black hole

We fabricate the waveguide arrays in borosilicate glass and implement the photonic lattices on a single chip with different evolution distances varying from 20 mm to 50 mm in a step of 5 mm. The required initial transverse wavevector is well achieved via a modulated array of waveguide. The heralded single photon is injected into the entry site in lattice and the outgoing probability distribution is measured at the output facet by single-photon imaging (see Methods for details).

**Figure 2.**
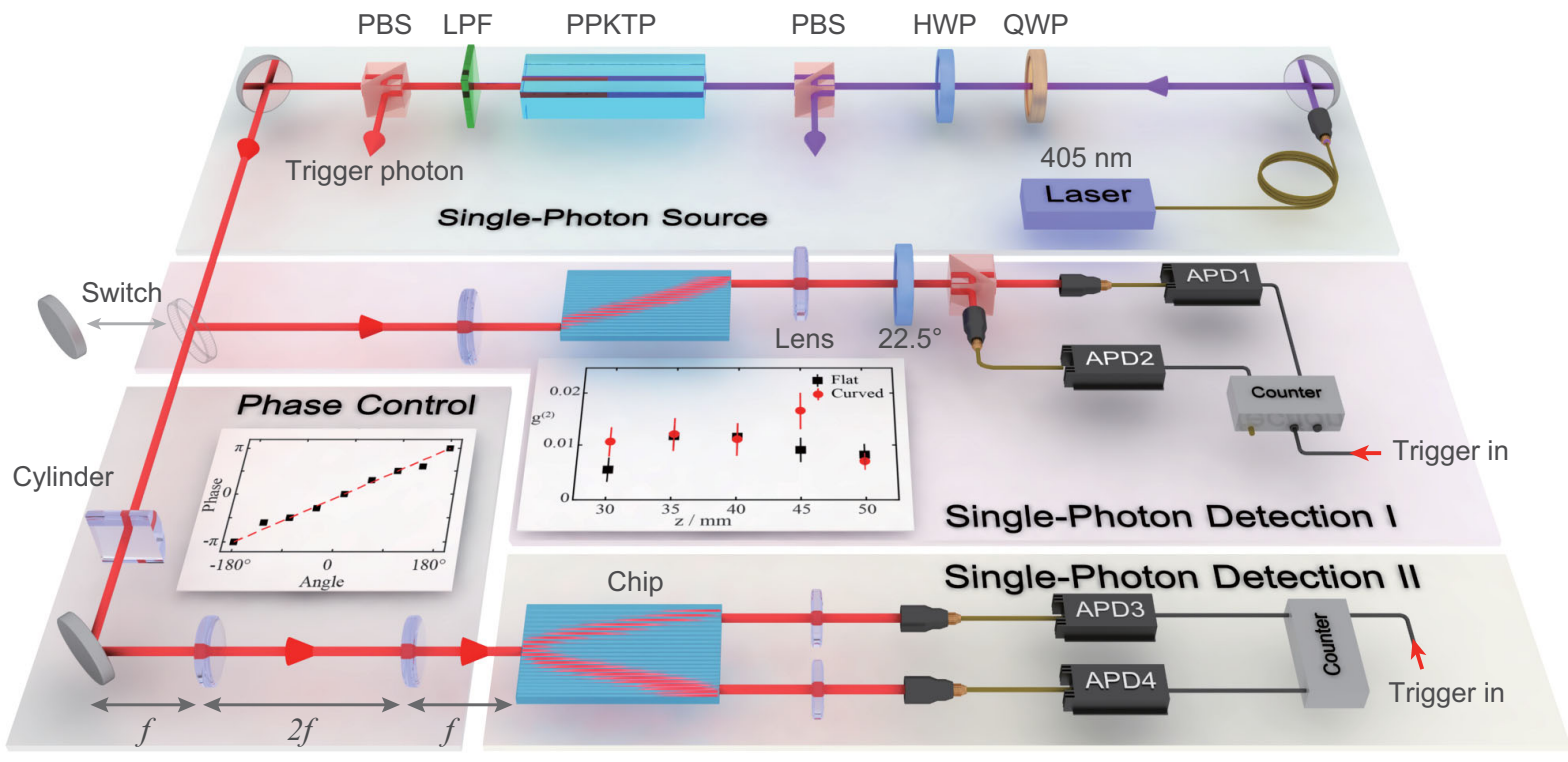
Experimental setup. The setup consists of three parts, including single-photon source, phase control and single-photon detection. The inset in the part of single-photon detection I shows the measured results of the second-order correlation function of single-photon wave packets at the output facet. The inset in phase control part shows the relationship between the phase of single-photon wave packet at the input facet of chip and the angle of the controller for the mirror. The well-matched linear relationship makes the phase of single-photon wave packet be conveniently controllable. The separation effect of fermion pair has the exact functioning that is required by Hanbury-Brown-Twiss effect. Both the wave packets with positive and negative energy states can be coupled into spatially different modes and register at different single photon detectors to observe the second-order correlation function *g*^(2)^.

Our quantum simulation setup, as shown in Fig. 2, consists of three parts, including single-photon generation, phase control, and single-photon detection. The single-photon generation of initial states associated with single-photon detection of the quantum evolution output ensures all the experimental investigations rigorously in quantum regime. Especially we employ Hanbury-Brown-Twiss effect to confirm the nonclassicality of evolved photonic states by measuring the heralded second-order correlation function *g*^(2)^. As shown in the inset of single-photon detection I, the experimental results of *g*^(2)^ along the evolution distance are all as low as 0.01, revealing strong nonclassicality and weak decoherence in the evolution process of quantum simulation.

**Figure 3.**
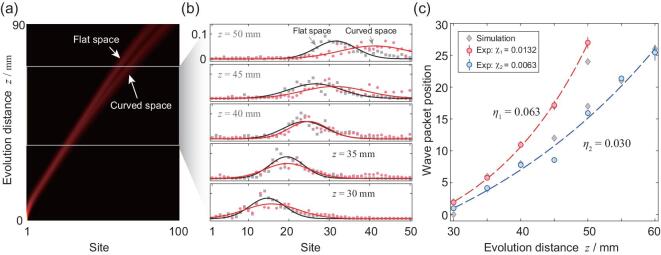
Experimental evolution of the single-photon wave packet near the event horizon of a black hole. (a) The combined results from the simulated results in flat and space around the black hole. The single-photon wave packet moves faster in the space around the black hole than that in flat space. (b) The measured probability distributions after different evolution distances varying from 30 to 50 mm. We fit the distribution with the Gaussian function as $P(x)=Ae^{(x-x_c)/2w^2}$ and set *x*_*c*_ as the positions of single-photon wave packets. The fitted results confirm the acceleration effect of single-photon wave packet in space near the event horizon of the black hole. (c) The position-time relationship of single-photon wave packet in the space of different black holes. The acceleration behavior of single-photon wave packet depends on the sizes of black hole, and the evolution index is inverse proportion to the sizes of black hole. The evolution function of the single-photon wave packet is fitted with *x*_*c*_ = *x*_0_*exp*(η · ($z$ − $z$_0_)) + *C*, and the initial evolution distance (time) $z$_0_ is 30, and evolution index η are 0.063±0.001 and 0.030±0.015 for the curvature of the black hole with 0.0132 and 0.0063, respectively.

The theoretical results of the single-photon probability distribution are shown in Fig. 3a. The single-photon Gaussian wave packets, corresponding to the boson, are found moving faster near the event horizon of the black hole than that in flat space in transverse direction. An Intensified Charge-coupled Device (ICCD) camera installed after the photonic chip is employed to observe the predicted deviation by recording the output probability distribution for different evolution distances (see Fig. 3b). By fitting the measured probability distributions with the Gaussian function, the differences between wave packets in flat space and space around the black hole appear more distinctly, which is well consistent with our theoretical prediction on the bosonic behavior near the event horizon of a black hole.

We further investigate the acceleration feature of the bosonic behavior in the analogue time domain by retrieving the fitted centric positions of single-photon wave packet (Fig. 3c). The position-time relation appears as exponential near the event horizon, presented as *x*_*c*_ = *x*_0_exp (η · ($z$ − $z$_0_)) + *C* with η_1_ = 0.063 ± 0.001, which clearly indicate the acceleration behavior as predicted by the theory. Such an acceleration should be determined by the curvature of the black hole. To demonstrate this, we fabricate another lattice with a different size of a black hole. The evolution function also appears as exponential but with a lower index η_2_ = 0.030 ± 0.015. We can obtain the ratio of the evolution indexes for two lattices as *q*_*e*_ = η_1_/η_2_ = 2.10 ± 1.02. The gradient parameters of coupling coefficient we selected in our lattices are χ_1_ = 0.0132 and χ_2_ = 0.0063, respectively, which lead to the curvature ratio of two lattices as *q*_*c*_ = χ_1_/χ_2_ = 2.10. The observed quantum evolution of single-photon wave packet demonstrates a distinct index dependence on the curvature of the designed lattices, suggesting a great match with our theoretical model on the dynamic behavior of boson in close proximity to the black hole well.

### The fermion pair generated by Hawking Radiation in close proximity to a black hole

Besides the dynamic behavior of boson, it is more inspiring to look into the evolution of fermion pair generated by Hawking Radiation in close proximity to a black hole, which is also possible to be experimentally investigated by designing artificial lattice structures on a photonic chip. We start with the Dirac equation under general relativity for a massless particle:

(3) \begin{equation*} \gamma ^{u} \nabla _{u} \varphi =0, \end{equation*}

where *u* is 0 for time and 1 for spatial 1 dimensional direction, ϕ = (*a*, *b*)^*T*^ (*T* stands for transpose) is a two-component spinor, γ^*u*^ are the Dirac gamma matrices, and the covariant derivative ∇_*u*_ = ∂_*u*_ + Ω_*u*_. Considering the metric of Schwarzschild black hole, there is a nonzero spin connection ω_010_ = −ω_100_ = α, thus Ω_0_ = −*i*ω_*cd*0_σ^*cd*^/4, Ω_1_ = 0, where σ^*cd*^ = *i*[γ^*c*^, γ^*d*^]/2. After choosing γ^0^ = σ_$z$_, γ^1^ = *i*σ_*y*_, the Eq. (3) can be written as: *i*∂_*t*_ϕ = *h*ϕ = −*i*α*r*σ_*x*_(∂_*r*_ + 1/2*r*)Φ, where *h* = −*i*α*r*σ_*x*_(∂_*r*_ + 1/2*r*) is Hamiltonian density (see Supporting Information II for details). The Hamiltonian has plane solutions, and the positive energy state *E*_+_ = α*k*_*r*_*r* has $\varphi _{+}(r, t)=({\scriptsize\begin{array}{@{}*{1}{c}@{}} {1}\\[0pt] {1} \end{array}})\exp \left(i k_{r} r+i \alpha k_{r} r t\right) / \sqrt{r}$, negative energy state *E*_−_ = −α*k*_*r*_*r* has $\varphi _{-}(r, t)=({\scriptsize\begin{array}{@{}*{1}{c}@{}} {1}\\[0pt] {-1} \end{array}})\exp \left(i k_{r} r-i \alpha k_{r} r t\right) / \sqrt{r}$, where *k*_*r*_ is the wavevector of the plane wave (see Supporting Information III for details).

In order to see the dynamic behavior of fermion pair with positive-negative energy solution close to the black hole, the time dependence position operator in the Heisenberg picture is written as *r*_*H*_(*t*) = *e*^*iht*^*re*^−*iht*^ = σ_*x*_sinh (α*t*)*r* + cosh (α*t*)*r*. We consider a Gaussian wave packet with positive energy state $\varphi _{+}(r, 0)=\frac{N}{\sqrt{2 r}} \int d k_{r} \cdot e^{-w^{2}\left(k_{r}-k_{0}\right)^{2} / 2+i k_{r} r}({\scriptsize\begin{array}{@{}*{1}{c}@{}} {1}\\[0pt] {1} \end{array}})$,  where *N* is normal coefficient, and the packet is centered at *k*_0_ and is characterized by a width of $w$. A direct calculation gives the evolution of the wave packet $\overline{r}_{+}(t)=$$\left\langle \varphi _{+}(r, 0)\left|r_{H}(t)\right| \varphi _{+}(r, 0)\right\rangle = r_{0} e^{\alpha t}$, where *r*_0_ is the initial position of wave packet. We can obtain the group velocity of this wave packet $\overline{v}_{+}(t)=$$d \overline{r}_{+} / d t=\alpha r_{0} e^{\alpha t}=\alpha \overline{r}_{+}$. For a Gaussian wave packet with negative energy $\varphi _{-}(r, 0)=\frac{N}{\sqrt{2 r}} \int d k_{r} \cdot e^{-w^{2}\left(k_{r}-k_{0}\right)^{2} / 2+i k_{r} r}({\scriptsize\begin{array}{@{}*{1}{c}@{}} {1}\\[0pt] {-1} \end{array}})$, the dynamic evolution is described as $\overline{r}_{-}(t)=r_{0} e^{-\alpha t}$, and the group velocity is $\overline{v}_{-}(t)=-\alpha r_{0} e^{-\alpha t}=-\alpha \overline{r}_{-}$. We can define the evolution distance between fermion pair as:

(4) \begin{equation*} s(t)=\overline{r}_{+}(t)-\overline{r}_{-}(t)=2 r_{0} \sinh (\alpha z) . \end{equation*}

By comparing the time evolution of wave packet with different energy, we can find that the positive energy state has positive velocity and escapes away from a black hole, and the negative state has negative velocity, propagating towards and eventually stopping around the black hole. The evolution process exhibits a good analogue with Hawking Radiation. Owing to vacuum fluctuations, particle-antiparticle pair is generated close to the event horizon of a black hole. One of the particles with negative energy falls into the black hole while the other escapes. This causes the black hole to lose mass to an outside observer, behaving that the black hole emits a particle.

### Experimental observation of the creation and evolution of fermion pair

**Figure 4.**
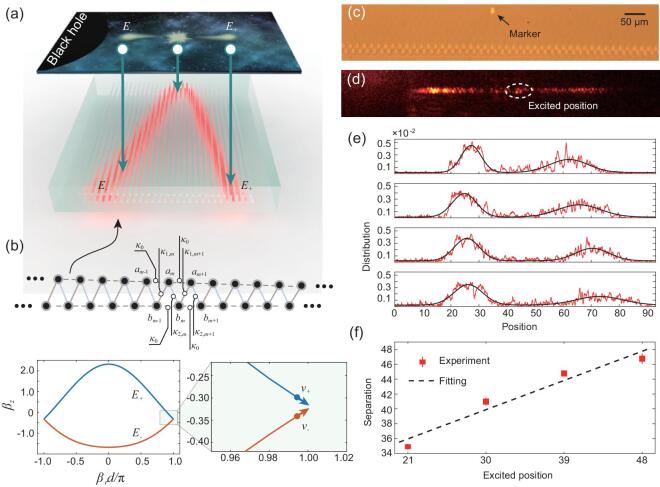
The creation and evolution of a fermion pair. (a) The schematic of mapping the behavior of fermion pair into a photonic lattice. The wave packets with negative and positive energy propagate into and away from the black hole, respectively. (b) The designed bi-layer waveguide lattice and the corresponding dispersion relation. The κ_0_ is adopted as 0.15 mm^−1^, β_0_ is uniform and is adopted as 0 in calculation, and κ_1_ is adopted as 1.0 mm^−1^. The inset of the dispersion relation shows the group velocity of positive (negative) energy state. (c) The cross-section profile of the fabricated lattices in a photonic chip. The marker is set to record the excited position. (d) The imagined output probability distribution of single-photon wave packet. The single-photon wave packet is split to two parts and they move in opposite direction. The white circle points out the excited position. (e) The output probability distributions with different excited positions in the same lattice. The site coordinates are relative values. (f) The separation distance increases with the excited position linearly. The fitted slope is 0.44±0.07.

To demonstrate the creation and evolution of fermion pair in close proximity to the black hole in the photonic lattice, we use bi-layer waveguide lattice to implement the two components of the Dirac equation (Fig. 4a). We firstly discrete the continuous Hamiltonian $\widehat{h}=-i \alpha r \sigma _{x}\left(\partial _{r}+1 / 2 r\right)$ in the photonic lattice and start with [54] $H=\int d r(\hat{h} \varphi )^{+} \varphi / 2+\int d r \varphi ^{+} \hat{h} \varphi / 2$, then obtain the discrete Hamiltonian $H=\frac{i}{2} \sum _{m} \alpha m(a_{m+1}^{+} b_{m}+a_{m}^{+} b_{m-1}-2 a_{m}^{+} b_{m}- b_{m}^{+} a_{m+1}- b_{m-1}^{+} a_{m}+2 b_{m}^{+} a_{m})$, where *m* and *d* are the number and distance of the photonic lattice, respectively (see Supporting Information IV and V for details). Finally, we obtain the coupling equation in the photonic lattice:

(5) \begin{equation*} \begin{array}{l}i \frac{d \tilde{a}_{m}}{d t}=\left[\tilde{a}_{m}, H\right]=-\alpha (m-1 / 2) \tilde{b}_{m-1}-\alpha m \tilde{b}_{m}, \\[10pt] {i \frac{d \tilde{b}_{m}}{d t}=\left[\tilde{b}_{m}, H\right]=-\alpha (m+1 / 2) \tilde{a}_{m+1}-\alpha m \tilde{a}_{m}},\nonumber\\[6pt] \end{array} \end{equation*}

where $\tilde{a}_{m}=i^{2 m} a_{m}$, $\tilde{b}_{m}=i^{2 m+1} b_{m}$. Therefore, we can construct bi-layer waveguide lattice in experiment with coupling coefficients between two layers as κ_1, *m*_ = α(*m* − 1/2), κ_2, *m*_ = α*m*, which both increase linearly with the photonic lattice site *m*, as shown in Fig. 4b. With the designed lattice, we can experimentally investigate the creation and evolution of fermion pair with positive and negative energy.

In the view of momentum space, the dispersion relation of the bi-layer lattice gives the key element of the successful mapping relation discussed above. Although the chain is not homogenous, we can define the local dispersion at the site *m* as $\beta _{z}^{m}=\beta _{0}-2 \kappa _{0} \cos \left(\beta _{r} d\right) \pm \sqrt{\left(\kappa _{1, m}-\kappa _{2, m}\right)^{2}+4 \kappa _{1, m} \kappa _{2, m} \cos ^{2}\left(\beta _{r} d / 2\right)}$; as shown in the inset in Fig. 4b, the dispersion relation changes from (i) to (ii) with the increase of κ_1, *m*_ and κ_2, *m*_ and maintains two modes. The separated two modes, corresponding to the two wave packet solutions of the system, are mapped to the fermion pair with positive and negative energy, respectively, as discussed above. The group velocity of positive (negative) energy of state is $v$_+_ ≅ α*m*($v$_−_ ≅ −α*m*), which corresponds to the analytical theoretical calculation based on Dirac equation well (see Supporting Information for details).

With femtosecond laser direct writing, we prototype the bi-layer lattices in a photonic chip and write additional waveguides in different depth to mark the excited position, as the facet of chip shown in Fig. 4c. The experimental setup is shown in Fig. 2, where the phase control part is in charge of preparing the required initial state and the part single-photon detection II is to confirm the experiment in the quantum regime (see Methods for details). From Fig. 4d, we can see that the prepared single-photon wave packet is split to two with the positive and negative energy states, respectively, possessing distinctly different evolution directions to either escape away or move towards the black hole.

We will be able to see the separation effect of fermion pair if we change the excited position *r*_0_ in the same lattice in light of Eq. (4). From the measured probability distributions of single-photon wave packet shown in Fig. 4e, we do observe that the separation distance changes with the excited position. We fit the probability distributions of single-photon wave packets with the bi-Gaussian function, $P(x)=A \mathrm{e}^{\left(x-x_{c 1}\right) / 2 \mathrm{w}^{2}}+B e^{\left(x-x_{c 2}\right) / 2 v^{2}}$ and define the separation distance as *s* = |*x*_*c*1_ − *x*_*c*2_|. We illustrate the obtained relationship between the separation distance and excited position in Fig. 4f, which follows a good linearity and is consistent with the theoretical calculation based on Eq. (4). In our experiment, the α is adopted as 0.005 mm^−1^, and $z$ is picked as 40 mm. The slope 2sinh (α$z$) is expected to be 0.40 in theory, while we get 0.44± 0.07 in experiment.

Very interestingly, the separation effect of fermion pair has the exact functioning that is required by Hanbury-Brown-Twiss effect. It means that there is a built-in Hanbury-Brown-Twiss interferometer for a fermion pair, which is intrinsically enabled by the quantum evolution of the pair itself. It would be very inspiring to extend experimental Hanbury-Brown-Twiss interference to the scenarios of both cosmological and quantum regime. While it will be never possible for people to detect the wave packet with negative energy falling into a black hole, both the wave packets with positive and negative energy states can be readily detected in our quantum simulation experiment. As is shown in single-photon detection II in Fig. 2, the separated wave packets can be coupled into spatially different modes and register at different single-photon detectors. Since the heralded single photon is the practical carrier for the positive and negative states of the fermion pair, the measurement of second-order correlation function *g*^(2)^ should show very strong nonclassicality. In our experiment, we observe the second-order correlation function *g*^(2)^ as low as 0.062±0.005, implying that single-photon nonclassicality can be well preserved in simulating the creation and evolution of a fermion pair close to the event horizon of an artificial black hole, and can be well observed by the built-in Hanbury-Brown-Twiss interferometer enabled by the quantum simulation phenomenon itself.

The built-in quantum interference predicted by the quantum field theory under general relativity can be observed in our quantum simulation platform, but it will never be possible to be observed with any cosmological approach. To our knowledge, it is the first quantum simulation being able to verify the predicted phenomena that have never been experimentally observed, beyond a stage of only repeating known phenomena. Furthermore, we emphasize that such quantum simulations can be even used to detect the quantum interference of fermion pair, which will be never possible to achieve in future experiments.

## CONCLUSION

In summary, we experimentally demonstrate quantum evolution of single-photon wave packet in close proximity to artificial black hole in evanescent coupling waveguides on a photonic chip. The simulation of the space near the event horizon of the black hole is achieved by precisely tuning the evanescent coupling between waveguides. We experimentally observe that the time evolution of single-photon wave packet around the black hole has an exponential acceleration form. Furthermore, we experimentally observe the creation and evolution of fermion pair near the event horizon: a wave packet of single photon with positive energy escapes from the black hole while negative energy is captured.

While it is still a big challenge in understanding the nature of gravity, especially in unifying general relativity and quantum mechanics, the quantum field theory related with the gravitational effect has been developed to bridge the two fields with new striking quantum phenomena predicted, such as Hawking radiation. Furthermore, we may question what quantum systems behave like if we could do experiments near a black hole, which are also impossible to be directly observed with nowadays technologies. Thus, it is very desirable to simulate these phenomena in quantum regime rather than classically, to cooperate with the advances of developing quantum gravity theory. We achieve this by demonstrating quantum evolution of genuine single photon rather than classical light in close proximity to an artificial black hole on a photonic chip. The gravitational field is the only artificial part in our quantum simulation platform, fortunately, and is accessible with cosmological observation.

In the current work, the static metric of two-dimensional Schwarzschild black hole is emulated by using one-dimensional evanescent coupling waveguides. Furthermore, we can construct more higher dimensional curved spacetime based on our experimental platform in future researches. For example, we can emulate three-dimensional spacetime using two-dimensional waveguide arrays, and emulate four-dimensional spacetime using two-dimensional waveguide arrays plus with an extra synthetic dimension using photons polarization or frequency. Besides, due to the propagation direction playing the role as the time in our experimental platform, dynamics metric can also be emulated based on this experimental platform, such as FRW metric, a model describing cosmic expansion as time evolution, and gravitational wave, which is the ripple of spacetime.

## METHODS

### Constructing an artificial black hole on a photonic chip

In our experimental implementation on observing quantum evolution of photon in close proximity to a black hole, the constructed photonic lattice consists of three parts. In the first part, we accurately modulate the coupling configuration among 10 sites with $\kappa _i=C\sqrt{(i(M-i))}$ to achieve the Gaussian wave packet, where *C* = 0.05, *M* = 19. A single-photon Gaussian wave packet with β_*r*_ = π/2*d* can be generated when only the first site is excited with single photon, which is key to faithfully satisfy the required transverse wavevector in theory. The second part is the transition section with 4 sites, and we set the coupling coefficients uniform as κ_9_. There are 36 sites in the last part, in which the coupling configuration implements the theoretical model of the space of event horizon and flat spaces, respectively.

### Fabrication of the photonic chip

We fabricate the photonic lattices according to the characterized coupling coefficients which are modulated by the separation between two adjacent waveguides. The lattices are prototyped in borosilicate glass (refractive index *n*_0_ = 1.514 for the direct write laser at 513 nm) by femtosecond laser direct writing. The laser owns 290 fs pulse duration and 1MHz repetition rate. We control the shape and size of the focal volume of the writing beam with a beam-shaping cylindrical telescope and then focus the laser beam into the borosilicate substrate with a 50× objective lens (NA 0.55). A high-precision three-axis motion stage is used to move the chip substrate during the fabrication with a constant velocity of 15 mm/s.

### The generation and imaging of the heralded single photon

We generate the single-photon pair at the wavelength of 810 nm via spontaneous parametric down conversion by pumping a periodically-poled KTP (PPKTP) crystal. The generated photon pair is separated to two components, horizontal and vertical polarization, after a long-pass filter and a polarized beam splitter (PBS). It should be noticed that, if we inject only one polarized photon into the lattices without externally triggering the ICCD with another polarized photon, the quantum evolution associated with the measured patterns would come from the thermal-state light rather than genuine single-photon state. Therefore, we inject the horizontally polarized photon into the lattices using a 20× objective lens, while the vertically polarized photon acts as the trigger for heralding the horizontally polarized photon out of the lattices with a time slot of 10 ns. We capture each evolution result using the ICCD camera behind a 10× microscope objective lens (not shown in Fig. 2) after accumulating in the external mode for 2000 s. The second-order anti-correlation parameter *g*^(2)^ is measured using avalanche photodiodes (APDs) and a Field-Programmable Gate Array (FPGA) counter after accumulating for 300 s.

### Experimental implementation on generating Gaussian wave packet and controlling the phase in bi-layer photonic lattices

We shape the heralded single-photon source with cylindrical lens to obtain an elliptical single-photon Gaussian input beam, and image its Fourier transform after a 20× lens on a rotatable mirror. A 4*f* system (two identical lenses at a distance of 2*f* from one another) is used to image the single-photon beam at the mirror into the lattices, which means that we can control the input angle of the beam by rotating the mirror while maintaining its shape. In this way, we are able to implement the required transverse wavevector (input phase) by controlling the input angle.

## Supplementary Material

nwaa111_Supplemental_File
