# Supplementary material for: Quantum simulation of particle pair creation near the event horizon
Source: Natl Sci Rev. 2020 May 30;7(9):1476–84. doi: 10.1093/nsr/nwaa111 (PMC8288817; doi:10.1093/nsr/nwaa111)
Supplement: nwaa111_Supplemental_File [file nwaa111_supplemental_file.pdf]

Supporting Information: Quantum Simulation of Particle Pair Creation near the Event Horizon

Yao Wang[1,*], Chong Sheng[2,*], Yong-Heng Lu[1], Jun Gao[1], Yi-Jun Chang[1], Xiao-Ling Pang[1], Tian-Huai Yang[1], Shi-Ning Zhu[2], Hui Liu[2,†], Xian-Min Jin[1,3,‡]

[1]Center for Integrated Quantum Information Technologies (IQIT), School of Physics and Astronomy and State Key Laboratory of Advanced Optical Communication Systems and Networks, Shanghai Jiao Tong University, Shanghai 200240, China
[2]National Laboratory of Solid State Microstructures & School of Physics, Collaborative Innovation Center of Advanced Microstructures, Nanjing University, Nanjing, Jiangsu 210093, China
[3]CAS Center for Excellence and Synergetic Innovation Center in Quantum Information and Quantum Physics, University of Science and Technology of China, Hefei 230026, China
[*]Y.W. and C.S. contributed equally to this work.
[†]E-mail: liuhui@nju.edu.cn
[‡]E-mail: xianmin.jin@sjtu.edu.cn

I. Constructing gravitational filed using photonic waveguide lattice with nonuniform coupling coefficients

Inspired by transformation optics, we use femtosecond laser written photonic waveguide lattice to mimic gravitational field and investigate the dynamic evolution of boson in close proximity of black hole. Firstly, we start by considering the line element of a two-dimensional Schwarzschild spacetime:

$$ds^2 = (1 - 2M/\rho)dt^2 - (1 - 2M/\rho)^{-1}dr^2 \tag{S1}$$

where M is the mass of black hole, and nature units have been adopted ($G = c = 1$). In our work, we are interested in the short distance behavior of Hawking radiation near the horizon ($\rho \approx 2M$), the Schwarzschild metric can be written as:

$$ds^2 = \alpha^2 r^2 dt^2 - dr^2 \tag{S2}$$

where $\alpha = 1/2r_s$, $r_s = 2M$, $r(\rho) = \sqrt{8M(\rho - 2M)}$. What is more interesting is that the Schwarzschild metric near the horizon of black hole has the same form as the line element of the Rindler [1].

While the quantum evolution of boson for null geodesic satisfies $ds = 0$, which means $|dr/r| = \alpha dt$, then we can obtain the evolution function of boson near to black hole as

$$r = r_0 e^{\alpha t}, \tag{S3}$$

where r_0 is initial position. The quantum evolution depends on the curvature of black hole α, which means that the quantum evolution is faster near the event horizon of a black hole with small radius.

For the spacetimes based on Eq. (S1), the evolution of boson similar to that in an optical media with an effective refraction index based on transformation optics [2–4] [Fig. S1(c)]:

$$n = \sqrt{-g_{11}/g_{00}} = 1/\alpha r, \tag{S4}$$

where $g_{00} = \alpha^2 r^2$, $g_{11} = -1$. In our work, we use evanescently coupled photonic waveguide lattice with designed coupling coefficient to achieve the required inhomogeneous effective refractive index. The dynamics of single-photon wave packet in photonic waveguide lattice can be described by a set of coupled discrete Schrodinger equations, which are derived from Schrodinger-type paraxial wave equation by employing the tight-binding approximation:

$$i\partial\varphi_m/\partial z = \beta_0\varphi_m - \kappa_m\varphi_{m-1} - \kappa_{m+1}\varphi_{m+1}, \tag{S5}$$

where φ_m is the complex field amplitude of site m, z is the propagation distance along the waveguides mapping the time variable, β_0 is on site energy of each waveguide, and parameter κ_m represents the coupling strength between the adjacent sites. Taking coupling coefficients as $\kappa_m = \kappa_{m+1} = \kappa$ and substituting the complex field amplitude with the plane wave solution $\varphi_m = A \cdot exp(i\beta_r md + i\beta_z z)$, we can obtain the dispersion connecting transverse and

longitudinal dynamics as [Fig. S1(b)] :

$$\beta_z = \beta_0 - 2\kappa \cos(\beta_r d), \tag{S6}$$

where A is the amplitude of plane wave, β_r and β_z are transverse and longitudinal wavevector respectively, d is waveguide spacing ($r = md$). After photon evolves in the such a waveguide over distance Δz, each transverse component gains a phase $\Phi = \beta_z(\beta_r)\Delta z$, and the corresponding transverse shift of a wave centered around β_r is $\Delta r = \partial\Phi/\partial\beta_r = (\partial\beta_z/\partial\beta_r)\,\Delta z$. Due to that the propagation distance z in the coupled waveguide equation plays the role as the time t in Schrodinger equation, hence when exciting the coupled waveguide with the transverse wavevector as around $\beta_r = \pi/2d$, we can define the velocity of wavepacket in such system as [Fig. S1(b)]:

$$v = \frac{\Delta r}{\Delta z} = \frac{\partial \beta_z}{\partial \beta_x} = 2\kappa d. \tag{S7}$$

And the refractive index of such photonic lattice is

$$n = 1/v = 1/2\kappa d. \tag{S8}$$

Comparing Eq. (S3) and (S7), to constructe gravitational field of $1{+}1$ dimensional Schwarzschild black hole in evanescently coupled waveguides, the coupling coefficients should satisfy

$$\boldsymbol{\kappa} = \alpha r/2d = \alpha m/2, \tag{S9}$$

where we take discrete the continuous function with lattice $r = md$. Therefore, by constructing the coupling coefficient κ increasing linearly with waveguide site m [Fig. S1(d)], we can achieve effective refractive index required by $1+1$ dimensional black hole. In Fig. S1(e) and Fig. S1(f), we show the dynamic evolution of single-photon wave packet in the flat space and near the event horizon of black hole, which clearly show the evolution has exponential form near the event horizon of black hole compared with linear evolution in the flat space.

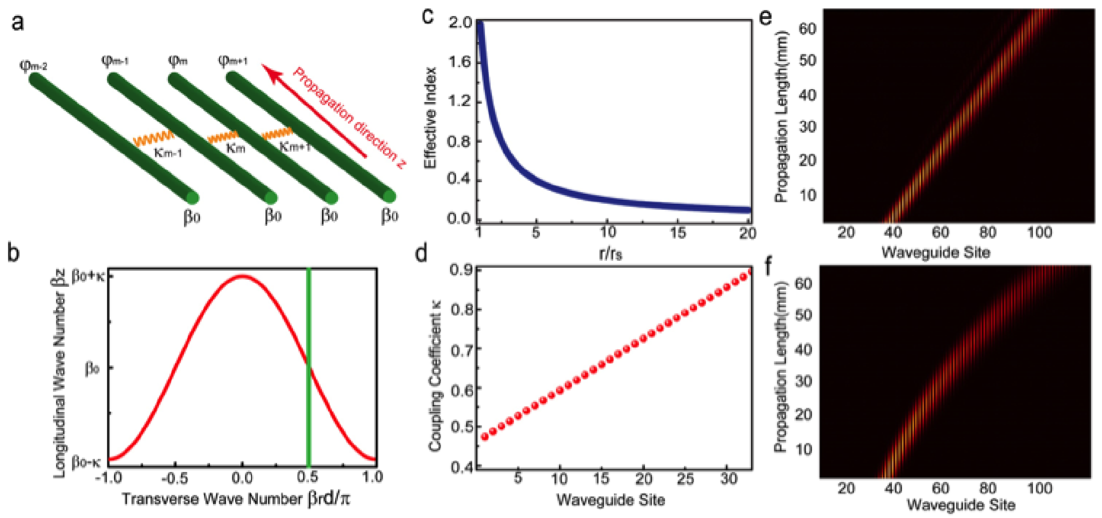

Figure S1: (a) The schematic of evanescently coupled waveguides. (b) The dispersion relation $\beta_z(\beta_r)$. The green line indicates the transverse wavevector as $\beta_r = \pi/2d$. (c) The effective index corresponds to $1+1$ dimensional Schwarzschild black hole given by transformation optics. (d) The corresponding coupling coefficient in evanescently coupled waveguides which can mimic the gravitational field of black hole. (e) The evolution of photon in the flat space. (f) The evolution of photon near the event horizon of black hole.

II. The Dirac equation near the event horizon of black hole

Besides the dynamic behavior of photon as boson, we can also investigate the evolution of fermion pair generated by Hawking Radiation in close proximity to black hole. We start with the Dirac equation in the $1+1$ dimensional spacetime for a massless particle is

$$\gamma^{\mu}\nabla_{\mu}\varphi = 0, \tag{S10}$$

where μ is 0 for time and 1 for spatial 1 dimensional direction, $\varphi = (a,b)^T$ (T stands for transpose) is a two-component spinor, γ^{μ} are the Dirac gamma matrices, and the covariant derivative $\Delta_{\mu} = \partial_{\mu} + \Omega_{\mu}$. Considering the metric Schwarzschild black hole according to

Eq. (S1), we obtain the element of metric as

$$\begin{aligned} g_{00} &= (\alpha r)^2, \\ g_{11} &= -1 \\ g^{00} &= 1/(\alpha r)^2, \\ g^{11} &= -1 \end{aligned} \tag{S11}$$

Then we can get the calculated Christoffel symbol as

$$\Gamma^i_{kl} = \frac{1}{2} g^{im} \left(\frac{\partial g_{mk}}{\partial x^l} + \frac{\partial g_{ml}}{\partial x^k} - \frac{\partial g_{kl}}{\partial x^m} \right), \tag{S12}$$

the Einstein convention is adopted here where the repeated indices are summed over. We have for nonzero Christoffel symbol as

$$\Gamma^0_{01} = \Gamma^0_{10} = 1/r, \quad \Gamma^1_{00} = \alpha^2 r. \tag{S13}$$

The calculated spin connection ω^a_{bv} can be directly obtained as

$$\omega^a_{bv} = e^a_\mu \partial_v \left(e^\mu_b \right) + e^a_\mu e^\sigma_b \Gamma^\mu_{\sigma v}, \tag{S14}$$

where e^a_μ (we use the convention that latin indices a, b are used to label local inertial coordinates and greek indices μ, v for general coordinates) is *vielbein* and satisfy the equation $e^\mu_a e^v_b g_{\mu v} = \eta_{ab}, e^a_\mu e^b_v \eta_{ab} = g_{\mu v}$, where $\eta_{ab} = diag(1, -1)$ is the Minkowski metric. Then we obtain $e^{\tilde{0}}_0 = 1/\alpha r, e^{\tilde{1}}_1 = 1$. Based on Eq. (S13), the nonzero spin connection is

$$\omega_{010} = -\omega_{100} = \alpha. \tag{S15}$$

Using the spin connection, we can obtain the calculated spinor $\Omega_v = \frac{1}{4}\omega_{abv}\sigma^{ab}$, where $\sigma^{ab} = \left[\gamma^a, \gamma^b\right]/2$. Then nonzero spinor is

$$\Omega_0 = \frac{1}{4}\omega_{010}\sigma^{01} + \frac{1}{4}\omega_{100}\sigma^{10} = \frac{\alpha}{4}\left[\gamma^0, \gamma^1\right]. \tag{S16}$$

Therefore, the Eq. (S9) can be written as

$$i\partial_t\varphi = -i\alpha r\gamma_0\gamma^1\partial_r\varphi - \frac{i\alpha}{4}\left[\gamma^0, \quad \gamma^1\right]\varphi. \tag{S17}$$

After choosing $\gamma^0 = \sigma_z$, $\gamma^1 = i\sigma_y$, the above equation can be simplified as

$$i\partial_t\varphi = h\varphi = -i\alpha r\sigma_x\partial_r\varphi - \frac{i\alpha}{2}\sigma_x\varphi, \tag{S18}$$

where h is Hamiltonian density.

III. The quantum evolution of single-photon wave packet in close proximity to an artificial black hole

In order to investigate the quantum evolution of single-photon wave packet near the event horizon of black hole, we assume that the Eq. (S17) has the solution $\varphi = \frac{1}{\sqrt{r}}\begin{pmatrix} a \\ b \end{pmatrix} e^{ik_r r - iEt}$, where k_r is the wavevector of the plane wave, E is the frequency of the plane wave, and then the Eq. (S17) can be written as

$$E\begin{bmatrix} a \\ b \end{bmatrix} = \alpha k_r r\begin{bmatrix} b \\ a \end{bmatrix}. \tag{S19}$$

Therefore, the solution has two energy state $E = \pm\alpha k_r r$. For positive energy state $E_+ = \alpha k_r r$, the solution is $\varphi_+(r,t) = \frac{1}{\sqrt{2r}}\begin{pmatrix} 1 \\ 1 \end{pmatrix} e^{ik_r r - iE_+ t}$; and for negative energy state $E_- = -\alpha k_r r$, the solution is $\varphi_-(r,t) = \frac{1}{\sqrt{2r}}\begin{pmatrix} 1 \\ 1 \end{pmatrix} e^{ik_r r - iE_- t}$.

In order to consider the dynamic behavior of single-photon wave packet with positive-negative energy solution close to the black hole, the time-depended position operator in the

Heisenberg pictures can be written as

$$
\begin{aligned}
r_H(t) &= e^{iht/\hbar} r e^{-iht/\hbar} \\
&= r + \frac{it}{h}\left(-i\hbar\alpha\sigma_x\right) r + \left(\frac{1}{2!}\right)\left(\frac{it}{h}\right)^2 \left(-i\hbar\alpha\sigma_x\right)^2 r \\
&\quad + \left(\frac{1}{3!}\right)\left(\frac{it}{h}\right)^3 \left(-i\hbar\alpha\sigma_x\right)^3 r \\
&\quad + \left(\frac{1}{4!}\right)\left(\frac{it}{h}\right)^4 \left(-i\hbar\alpha\sigma_x\right)^4 r + \ldots \\
&\quad + \left(\frac{1}{n!}\right)\left(\frac{it}{h}\right)^n \left(-i\hbar\alpha\sigma_x\right)^n r + \ldots \\
&= \left[1 + \frac{1}{2!}(\alpha t)^2 + \frac{1}{4!}(\alpha t)^4 + \frac{1}{2k!}(\alpha t)^{2k}\right] r \\
&\quad + \sigma_x \left[\alpha t + \frac{1}{3!}(\alpha t)^3 + \frac{1}{(2k+1)!}(\alpha t)^{2k+1}\right] r \\
&= \sigma_x \sinh(\alpha t) r + \cosh(\alpha t) r.
\end{aligned}
\tag{S20}
$$

We consider a single-photon Gaussian wave packet of positive energy state as

$$
\begin{aligned}
\varphi_+(r,0) &= N \int dk_r \cdot \frac{1}{\sqrt{2r}} e^{-w^2(k_r-k_0)^2/2} e^{ik_r r} \begin{pmatrix} 1 \\ 1 \end{pmatrix} \\
&= \frac{N\sqrt{\pi}}{w} \frac{1}{\sqrt{r}} e^{-\frac{r^2}{2w^2}} e^{ik_0 r^2} \begin{pmatrix} 1 \\ 1 \end{pmatrix},
\end{aligned}
\tag{S21}
$$

where N is normal coefficient, and the packet centered at k_0 is characterized by a width of w. A direct calculation gives the evolution of the wave packet as

$$
\begin{aligned}
\overline{r}_+(t) &= \left\langle \varphi_+(r,0) \left| r_H(t) \right| \varphi_+(r,0) \right\rangle \\
&= \left\langle \varphi_+(r,0) \left| \sigma_x \sinh(\alpha t) r + \cosh(\alpha t) r \right| \varphi_+(r,0) \right\rangle \\
&= \frac{N^2 \pi^{3/2}}{2w} e^{\alpha t} \\
&= r_0 e^{\alpha t}, \\
\overline{v}_+(t) &= \partial \overline{r}_+(t)/\partial t = \alpha \overline{r}_+(t).
\end{aligned}
\tag{S22}
$$

The single-photon Gaussian wave packet of negative energy state is

$$\begin{aligned}\varphi_-(r,0) &= N\int dk_r \cdot \frac{1}{\sqrt{r}} e^{-w^2(k_r-k_0)^2/2} e^{ik_r r}\begin{pmatrix}1\\-1\end{pmatrix}\\ &= \frac{N\sqrt{\pi}}{w}\frac{1}{\sqrt{r}} e^{-\frac{r^2}{2w^2}} e^{ik_0 r}\begin{pmatrix}1\\-1\end{pmatrix},\end{aligned} \tag{S23}$$

and the dynamic evolution can be described as

$$\begin{aligned}\overline{r}_-(t) &= \langle \varphi_-(r,0)\left| r_H(t)\right| \varphi_-(r,0)\rangle\\ &= \langle \varphi_-(r,0)\left|\sigma_x \sinh(\alpha t) r + \cosh(\alpha t) r\right| \varphi_-(r,0)\rangle\\ &= \frac{N^2\pi^{3/2}}{2w} e^{-\alpha t}\\ &= r_0 e^{-\alpha t},\end{aligned} \tag{S24}$$

$$\overline{v}_-(t) = \partial\overline{r}_-(t)/\partial t = -\alpha\overline{r}_-(t).$$

By comparing the time evolution of wave packet with different energy, we obtain that the positive energy state has positive velocity and exponentially escapes away from black hole, while the negative state propagates toward and eventually stops around the black hole. The evolution process is analogy of Hawking Radiation. Owing to vacuum fluctuations, particleantiparticle pair is generated close to the event horizon of a black hole. One of the particles with negative energy falls into the black hole while the other escapes. In order to preserve total energy, the particle that fell into the black hole must have had a negative energy (with respect to an observer far away from the black hole). This causes the black hole to lose mass to an outside observer; it would appear that the black hole has just emitted a particle.

IV. Discrete Dirac equation near the event horizon of black hole in the waveguide lattice

In order to discrete the continuous Hamiltonian in the photonic lattice, we start single-particle Hamiltonian h with [5]:

$$H = \frac{1}{2}\int dr (h\varphi)^{+}\varphi + \frac{1}{2}\int dr \varphi^{+} h\varphi. \tag{S25}$$

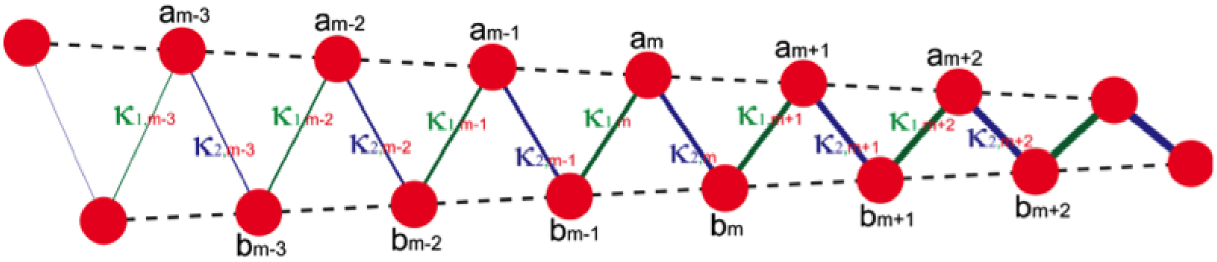

Figure S2: The schematic of optical lattice. a_m and b_m respectively corresponds to waveguide in the upper layer and lower layer, β_0 is on site energy of each waveguide, κ_0 is the nearest neighbor coupling coefficient in same layer, κ_{1m} and κ_{2m} are coupling coefficient between the upper and lower layer.

Considering two-component spinor $\varphi = (a, b)^T$, the above equation is written as:

$$H = \frac{i}{2} \int dr\alpha r \left(\partial_r a^+ b - a^+ \partial_r b \right) + h.c \tag{S26}$$

Furthermore, we discrete the continuous function with lattice as shown Fig. S2:

$$\begin{aligned} r &= md, \\ \partial_r a^+ &= \frac{a^+_{m+1} - a^+_m}{d}, \\ \partial_r b &= \frac{b_m - b_{m-1}}{d}. \end{aligned} \tag{S27}$$

The discrete Hamiltonian is

$$H = \frac{i}{2} \sum_m \begin{array}{l} \alpha m \left(a^+_{m+1} b_m + a^+_m b_{m-1} - 2a^+_m b_m \right. \\ \left. -b^+_m a_{m+1} - b^+_{m-1} a_m + 2b^+_m a_m \right) \end{array} . \tag{S28}$$

Then we obtain coupling equation in the optical lattice:

$$\begin{array}{l} i\frac{da_m}{dt} = [a_m, H] = i\alpha(m - 1/2)b_{m-1} - i\alpha m b_m, \\ i\frac{db_m}{dt} = [b_m, H] = -i\alpha(m + 1/2)a_{m+1} + i\alpha m a_m. \end{array} \tag{S29}$$

After taking $\tilde{a}_m = i^{2m} a_m$, $\tilde{b}_m = i^{2m+1} b_m$, the above equation is:

$$\begin{array}{l} i\frac{d\tilde{a}_m}{dt} = -\alpha(m - 1/2)\tilde{b}_{m-1} - \alpha m \tilde{b}_m, \\ i\frac{db_m}{dt} = -\alpha(m + 1/2)\tilde{a}_{m+1} - \alpha m \tilde{a}_m. \end{array} \tag{S30}$$

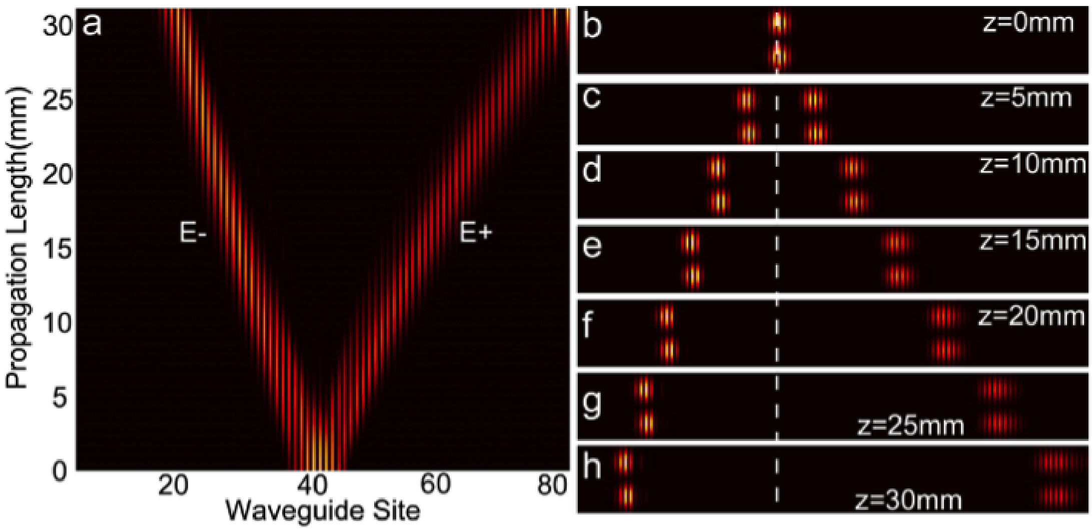

Figure S3: (**a**)The evolution of fermion pair with positive (E_+) and negative (E_-) energy state. (**b**)-(**h**)The intensity of waveguide section at different time

V. The inhomogeneous coupling between bi-layer waveguide

Furthermore, if we construct bi-layer lattice fabricated by femtosecond laser written technology as shown in Fig. S2, considering the nearest neighbor, the coupled wave equation can be described as:

$$\begin{array}{l} i\frac{da_m}{dz} = \beta_0 a_m - \kappa_0 \left(a_{m+1} + a_{m-1}\right) - \kappa_{1m} b_{m-1} - \kappa_{2m} b_m, \\ i\frac{db_m}{dz} = \beta_0 b_m - \kappa_0 \left(b_{m+1} + b_{m-1}\right) - \kappa_{1m} a_{m+1} - \kappa_{2m} a_m, \end{array} \tag{S31}$$

where a_m and b_m respectively corresponds to waveguide site in the upper layer and lower layer, β_0 is on site energy of each waveguide, κ_0 is the nearest neighbor coupling coefficient in same layer, κ_{1m} and κ_{2m} are coupling coefficient between the upper and lower layer. When assuming the approximated solution $\left(\begin{array}{c} a_m \\ b_m \end{array}\right) = \left(\begin{array}{c} A\exp\left(i\beta_r md - i\beta_z z\right) \\ B\exp\left(i\beta_r md - i\beta_z z\right) \end{array}\right)$ in local lattice, then we obtain $a_{m\pm1} = \exp(\pm i\beta_r d)a_m$, $b_{m\pm1} = \exp(\pm i\beta_r d)b_m$, the above equation can be written as:

$$\begin{array}{l} i\frac{da_m}{dz} = \left(\beta_0 - 2\kappa_0 \cos\left(\beta_r d\right)\right) a_m - \kappa_{1m} b_{m-1} - \kappa_{2m} b_m, \\ i\frac{db_m}{dz} = \left(\beta_0 - 2\kappa_0 \cos\left(\beta_r d\right)\right) b_m - \kappa_{1m} a_{m+1} - \kappa_{2m} a_m. \end{array} \tag{S32}$$

If assuming $\begin{pmatrix} \tilde{a}_m \\ \tilde{b}_m \end{pmatrix} = \begin{pmatrix} a_m \exp\left[-\left(\beta_0 - 2\kappa_0 \cos\left(\beta_r d\right)\right) z\right] \\ b_m \exp\left[-\left(\beta_0 - 2\kappa_0 \cos\left(\beta_r d\right)\right) z\right] \end{pmatrix}$, the above equation can be written as:

$$\begin{aligned} i\frac{d\tilde{a}_m}{dz} &= -\kappa_{1m}\tilde{b}_{m-1} - \kappa_{2m}\tilde{b}_m, \\ i\frac{d\tilde{b}_m}{dz} &= -\kappa_{1m}\tilde{a}_{m+1} - \kappa_{2m}\tilde{a}_m. \end{aligned} \tag{S33}$$

By comparing the Eq. (S29) and Eq. (S32), if we manipulate the coupling coefficient $\kappa_{1m} = \alpha(m-1/2)$, $\kappa_{2m} = \alpha m$ depending on waveguide site m, the quantum evolution of massless fermion pair with positive and negative energy generated by black hole can be emulated by the designed bi-layer optical lattice. And in the photonic lattice the propagation distance z plays the role as the time t, longitudinal wavevector β_z plays the role as the energy E, transverse wavevector β_r plays the role as the wavevector k_r of the Dirac fermion. Fig. S3 shows the evolution of fermion near the event horizon of black hole according to number calculation based on Eq. (S30). There are two branches which respectively corresponds to fermion with positive and negative energy as shown in Fig. S3(a): The positive energy state exponentially escapes away from black hole, and negative state propagates toward and eventually stops around the black hole. Fig. S3(b)-Fig. S3(h) shows the intensity of waveguide section at different time where the propagation distance plays as role of time in the waveguide.

In order to clearly clarify the positive and negative state in the waveguide, the dispersion relation at the site m in the momentum space according to Eq. (S30) can be given as:

$$\begin{aligned} \beta_z^m =& \beta_0 - 2\kappa_0 \cos\left(\beta_r d\right) \\ &\pm \sqrt{\left(\kappa_{1m} - \kappa_{2m}\right)^2 + 4\kappa_{1m}\kappa_{2m}\cos^2\left(\beta_r d/2\right)} \end{aligned} \tag{S34}$$

In Fig. S4, we show the two modes maintain separated with the change of parameters κ_{1m} and κ_{2m}.

After taking adiabatic approximation and assuming $\kappa_{2m} - \kappa_{1m} = \alpha/2 \ll \kappa_{1m}(\kappa_{2m})$ and $\kappa_{1m} \approx \kappa_{2m} = \alpha m$, the Eq. (S33) can be written as $\beta_z^m = \beta_0 - 2\kappa_0 \cos\left(\beta_r d\right) \pm 2\alpha m \cos\left(\beta_r d/2\right)$. Due to translational symmetry in the propagation, the β_z is a conserved quantity. In the initial

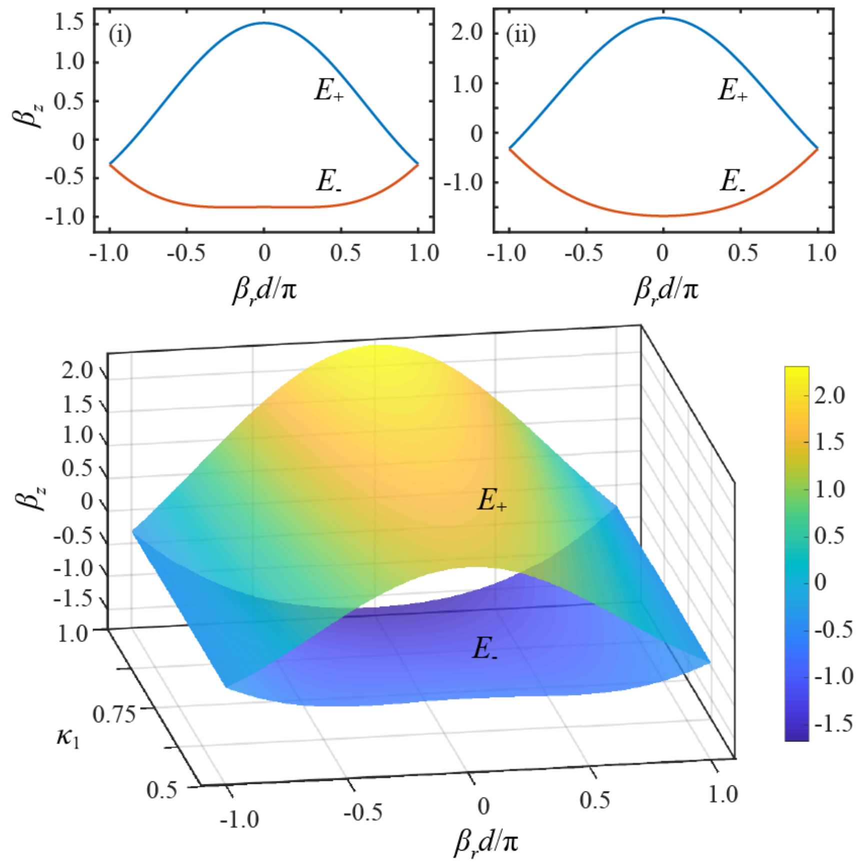

Figure S4: The spectrum of the bi-layer waveguide lattice. The two modes maintain separated with the change of parameters κ_{1m}. The inset (i) and (ii) give the dispersion relation of the fabricated bi-layer lattice, where κ_0 is adopted as $0.15\ mm^{-1}$, β_0 is uniform and is adopted as 0 in calculation, κ_1 is adopted as $0.5\ mm^{-1}$ for inset (i) and $1.0 mm^{-1}$ for inset (ii) respectively, and $\kappa_2 = \kappa_1 + 0.005 mm^{-1}$. For the whole lattice, κ_1 varies from 0.5 to $1.0\ mm^{-1}$ in step of $0.005 mm^{-1}$.

time of the exciting waveguide, β_z is taken as $\beta_z = \beta_0 - 2\kappa_0$. Then the group velocity of positive and negative wave packet is:

$$\begin{aligned} v_+ &= \partial\beta_z^+/\partial\beta_r = \alpha m, \\ v_- &= \partial\beta_z^-/\partial\beta_r = -\alpha m. \end{aligned} \tag{S35}$$

The Eq. (S34) is corresponding to Eq. (S21) and Eq. (S23) well. All these equations unveil that the positive energy state escapes away from black hole with increasing positive velocity, while the negative energy state propagates toward to black hole with decreasing negative velocity.

References

[1] Crispino L. C. B., Higuchi A., & Matsas G. E .A. The unruh effect and its applications. *Reviews of Modern Physics* **80**, 787–838 (2008).

[2] Pendry J. B., Schurig D., & Smith D. R. Controlling electromagnetic fields. *Science* **312**, 1780–1782 (2006).

[3] Leonhardt U. Optical conformal mapping. *Science* **312**, 1777–1780 (2006).

[4] Genov D. A., Zhang S., & Zhang X. Mimicking celestial mechanics in metamaterials. *Nature Physics* **5**, 687–692 (2009).

[5] Boada O., Celi A., Latorre J. I., & Lewenstein M. Dirac equation for cold atoms in artificial curved spacetimes. *New Journal of Physics* **13**, 035002 (2011).
